# Pregnancy in end-stage renal disease patients on hemodialysis: two case reports

**DOI:** 10.4076/1757-1626-2-8139

**Published:** 2009-08-12

**Authors:** Rohina Swaroop, Raja Zabaneh, Nakul Parimoo

**Affiliations:** Northwest Louisiana Nephrology L.L.C1800 Buckner Street, Suite C-120, Shreveport, LA 71101USA

## Abstract

**Introduction:**

Pregnancy in patients with end-stage renal disease is rare due to numerous factors that impair fertility. Even if pregnancy does occur pregnancy outcome with a live birth has a low success rate.

**Case presentation:**

We report two cases of successful pregnancy in patients with end-stage renal disease on hemodialysis.

**Conclusion:**

The purpose of hemodialysis is not only to maintain life but also to make quality of life as normal as possible for the end-stage renal disease patient. Propagation of life is basic to all life forms and the ability to do so can be considered as a success in a patient with end-stage renal disease.

## Introduction

Pregnancy in patients with advanced chronic kidney disease and patients with end-stage renal disease (ESRD) is rare, due to a multiple factors. We present two cases: a 33-year-old African American female and a 28-year-old African American female whose pregnancies were successfully managed.

Management of these patients requires a multi-disciplinary team approach - including obstetrician, hemodialysis or peritoneal dialysis unit staff, renal dietitian, nephrologists.

## Case presentation

### Case report 1

A 33-year-old African American female, gravida 4, with no abortions in the past and three living offspring, other medical problems included hypothyroidism. Etiology of ESRD was felt to be hypertensive nephrosclerosis secondary to poorly controlled hypertension. The patient had initiated hemodialysis in March 2004 and was receiving three times a week maintenance HD with no significant problems. Vascular access was a right internal jugular tunneled catheter. Six months after initiating hemodialysis, patient reported approximately 6 weeks of amenorrhea. She was found to be pregnant after measurement of serum HCG and pelvic ultrasound.

Hemodialysis prescription was changed to 6 days a week, three and a half hour per treatment. Dietary regime was liberalized in regards to protein consumption; serum albumin was maintained between 3.8-4 g/dl. Weekly Kt/V was maintained at a value between 6-8. (Kt/V is defined as the dialyzer clearance of urea (K), obtained from the manufacturer in ml/min, multiplied by the duration of the dialysis treatment (t, in minutes) divided by the volume of distribution of urea in the body (V, in ml)). She also received multivitamin and folic acid throughout the pregnancy. Hypertension remained a concern during this period, she was on an ACE-inhibitor prior to the pregnancy, this had to be discontinued, and she was treated with a regimen of hydralazine, labetalol, and alpha methyl dopa. Her BP averaged 110-140/70-90. Patient’s hemoglobin was maintained between 9-11 with use of Epogen administered three times a week, she was also given iron supplementation to maintain iron saturation of 20% or greater per the hemodialysis unit’s anemia and iron protocol.

Caesarean section was performed at 27^3^/_7_ weeks of gestation for non reassuring fetal status and malpresentation (transverse lie). She delivered a 1 pound 14 ounce female infant with an uneventful neonatal period. A year after, both mother and infant were in excellent condition.

### Case report 2

A 28-year-old black female with one living offspring and no abortions with past medical history of polycystic kidney disease, HTN, anemia of chronic disease and goiter. The patient had progressed to Chronic Kidney Disease Stage V and it was recommended that she initiate hemodialysis, however she had been refusing hemodialysis. She presented with abdominal distension and amenorrhea was found to be 12 weeks pregnant, diagnosed by serum HCG testing and pelvic ultrasound. Patient agreed to undergo hemodialysis once pregnancy was confirmed. Folate and Iron supplementation was started. Hemodialysis was initiated via tunneled dialysis catheter, 6 days a week, with each session 3 hours and 15 minutes duration. Weekly Kt/V was maintained at a value between 6-8. Dietary regime also was liberalized in regards to protein intake; serum albumin was maintained between 3.8-4 g/dl. Epogen was administered to maintain hemoglobin between 10-11 mg/dl. Iron supplementation was used to maintain iron saturation of 30% or greater per the hemodialysis unit’s anemia and iron protocol. BP was maintained between 110-140/70-80 mmHg using amlodipine, alpha-methyldopa, and labetalol. Patient was also administered oral multivitamins and folic acid.

At 29 weeks the obstetric team found that the fetal growth was lagging behind and the Doppler studies of the umbilical arteries were abnormal. Contraction stress test was found to be positive and she was delivered by Caesarean section resulting in a single viable female child weighing one and a half pound with an uneventful neonatal period. Patient and child remain healthy one year after.

## Discussion

In 1971 Confortini *et al.* [1] reported the 1st successful pregnancy in a 35 year old woman on chronic hemodialysis.

The largest study ‘Registry of pregnancy in dialysis patients’ showed 2% of patients on dialysis became pregnant over a 4-year period [2].The estimated frequency of conception in patients on dialysis is within a range as variable as 1.4% per year in Saudi Arabia to 0.5% in USA [3].

The reduced fertility is due to anovulation and hyperprolactinemia leading to oligomenorrhoea seen in female patients on dialysis [4]. Other factors that contribute are reduced libido due to altered human chorionic gonadotropin pulses and reduced renal leptin clearance [5,6]. Leptin levels are known to be high in obese women as well and may be a contributory factor to the infertility observed in them. Leptin has been shown to affect the hypothalamic-pituitary axis through neuropeptide Y and high affinity binding sites have been detected in the hypothalamus.

Even if pregnancy does occur in a patient with chronic kidney disease a study done in Japan [7] showed that it resulted in spontaneous abortion in 56% of patients, 11% developed still births, 14% had neonatal deaths, 18% had therapeutic abortion, approximately 40% abortions occurred in 2^nd^ trimester.

The outcome of pregnancies in such patients has markedly improved from approximately 20% live births during the 1980s [8] to 85% surviving infants according to case report published in Turkey in 2004 [9]. It has been shown that that the prognosis for successful conclusion of pregnancy is better for patients who begin dialysis after the onset of pregnancy as compared to patients who are already on dialysis (72.6% and 37.5%), respectively [2]. Our article reports cases representing both these groups.

An increase dose of dialysis with a weekly Kt/V of 6-8 or dialysis 5-6 days/week is considered beneficial [10]. We were also dialyzing both patients 6 days a week.

In 1997 recommendations had been published for the most appropriate treatment for patients on dialysis [4] listed in Table 1. These were the basis for treatment in both patients.

**Table 1.** Recommendations for treatment of patients on dialysis

1. Dialysis regime must maintain blood urea levels at 17 mmol/L (102.4 mg/dl).
2. If treating with hemodialysis, 5-7 sessions per week should be carried out, with minimum heparinization and low ranges of ultrafiltration.
3. If treating with peritoneal dialysis, reduce the volumes of dialysis solution (1.5 L) and increase the frequency.
4. Adapt the amount of calories and proteins: protein ingestion: 1 g/kg/day, adding 20 g/day for fetal growth. Add supplements of water-soluble vitamins and zinc.
5. Treatment of hypertension must be done under strict supervision and with pharmacological adaptation.
6. Correct anemia based on guidelines for the management of anemia in renal disease patients as per NKF-DOQI guidelines, reinforcing therapy with erythropoietin (compatible with proper blood pressure control) to keep hemoglobin above 10 g/dl and transferrin saturation above 30%.
7. Prevent metabolic acidosis.
8. Manage mineral metabolism; avoid hypo- and hypercalcemia.
9. Prevent hypomagnesemia with adequate dialysis baths and eventually with oral supplements.
10. Treat premature start of labor with beta-agonists and magnesium sulfate. Reinforce fetal monitoring, especially during hemodialysis sessions.

## Conclusion

Women on dialysis who wish to conceive or continue their existing pregnancies should be given special antenatal and neonatal care. Joint efforts of nephrologists, dialysis unit staff, nutritionists and obstetricians can help to make the pregnancy successful. Careful attention need to be paid to dialysis strategy, anemia control, fluid balance, control of hypertension and nutrition. It is appropriate that these patients should be managed in a high risk pregnancy facility including a neonatal intensive care unit. In patients who have already completed their family contraception should be advised as pregnancy can occur even after many years of being on dialysis.

## Abbreviations

ESRD: end-stage renal disease
NKF-K/DOQI: National Kidney Foundation Kidney Diseases Outcomes Quality Initiative
